# Field effectiveness of highly pathogenic avian influenza H5N1 vaccination in commercial layers in Indonesia

**DOI:** 10.1371/journal.pone.0190947

**Published:** 2018-01-10

**Authors:** Simson Tarigan, Michael Haryadi Wibowo, Risa Indriani, Sumarningsih Sumarningsih, Sidna Artanto, Syafrison Idris, Peter A. Durr, Widya Asmara, Esmaeil Ebrahimie, Mark A. Stevenson, Jagoda Ignjatovic

**Affiliations:** 1 Indonesian Research Centre for Veterinary Science, Bogor, Indonesia; 2 Faculty of Veterinary Science, Universitas Gadjah Mada, Yogyakarta, Indonesia; 3 Directorate General of Livestock and Animal Health Services, Jakarta, Indonesia; 4 CSIRO Australian Animal Health Laboratory, Geelong, Victoria, Australia; 5 School of Information Technology and Mathematical Sciences, Division of Information Technology, Engineering and the Environment, University of South Australia, Adelaide, South Australia, Australia; 6 School of Veterinary and Agricultural Sciences, University of Melbourne, Melbourne, Victoria, Australia; Sun Yat-Sen University, CHINA

## Abstract

Although vaccination of poultry for control of highly pathogenic avian influenza virus (HPAIV) H5N1 has been practiced during the last decade in several countries, its effectiveness under field conditions remains largely unquantified. Effective HPAI vaccination is however essential in preventing incursions, silent infections and generation of new H5N1 antigenic variants. The objective of this study was to asses the level and duration of vaccine induced immunity in commercial layers in Indonesia. Titres of H5N1 haemagglutination inhibition (HI) antibodies were followed in individual birds from sixteen flocks, age 18–68 week old (wo). The study revealed that H5N1 vaccination had highly variable outcome, including vaccination failures, and was largely ineffective in providing long lasting protective immunity. Flocks were vaccinated with seven different vaccines, administer at various times that could be grouped into three regimes: In regime A, flocks (n = 8) were vaccinated two or three times before 19 wo; in regime B (n = 2), two times before and once after 19 wo; and in regime C (n = 6) three to four times before and two to three times after 19 wo. HI titres in regime C birds were significantly higher during the entire observation period in comparison to titres of regime A or B birds, which also differed significantly from each other. The HI titres of individual birds in each flock differed significantly from birds in other flocks, indicating that the effectiveness of field vaccination was highly variable and farm related. Protective HI titres of >4log_2_, were present in the majority of flocks at 18 wo, declined thereafter at variable rate and only two regime C flocks had protective HI titres at 68 wo. Laboratory challenge with HPAIV H5N1 of birds from regime A and C flocks confirmed that protective immunity differed significantly between flocks vaccinated by these two regimes. The study revealed that effectiveness of the currently applied H5N1 vaccination could be improved and measures to achieve this are discussed.

## Introduction

Following the incursion of highly pathogenic avian influenza (HPAI) H5N1 virus into Indonesia in 2003, the disease spread rapidly throughout most of the country wherein it has become endemic [1]. As well as economic losses due to high mortality in poultry, there were also human fatalities through direct contact with infected poultry [2, 3]. As a result of the failure of a culling strategy to control the spread of the disease, a program of vaccination of backyard and commercial poultry against H5N1 was introduced in Indonesia in 2004 [1, 4, 5]. In accordance with the then international guidelines [4] heterologous H5N2 vaccines were used initially [6]; However, these were found to be suboptimal and subsequently vaccines developed from local H5N1 strains were approved [7, 8].

In Indonesia since 2003, a large number of H5N1 strains have been isolated [9, 10]. Sequencing of the major protein haemagglutinin (HA) and antigenic typing using the haemagglutination inhibition (HI) test have shown antigenic differences between strains that have emerged over time [8, 10, 11]. Early Indonesian H5N1 strains differed little by sequencing and are considered to have originated from a single introduction of H5N1 into the country in 2003 [12, 13]. All isolates belonged to subclade 2.1.1, of which the strain A/chicken/Legok/2003 H5N1was used for production of a vaccine in 2005 [7, 8, 14, 15]. By 2008 antigenic variants have emerged that belonged to subclades 2.1.2 and 2.1.3 of which A/chicken/wj/Pwt-Wij/2006 (Pwt) H5N1was developed into a vaccine since A/chicken/Legok/2003-based vaccine did not protect against Pwt challenge [8]. Recently, a new H5N1 variant A/duck/Sukoharjo/BBVW-1428-9/2012 (Skh) has emerged that belongs to a new subclade of H5N1 2.3.2.1 [16].

Laboratory studies have shown that vaccination with inactivated oil emulsion avian influenza (AI) vaccines has multiple beneficial effects including prevention of clinical signs and mortalities, reduction in the number of infected birds and consequently a reduction in the reservoir of virus in the environment [5, 6, 17, 18]. Factors that influence vaccination outcome include the type and quality of vaccine, vaccination schedule, the dose and method of administration [19]. Importantly there is no single recommended regime for HPAI vaccination of commercial poultry in the endemic situation [18, 20]. Vaccine induced immunity is measured by the presence of haemagglutination inhibiting (HI) antibodies in vaccinated birds and HI titres generally reflect the efficacy of the vaccine and correlate with protection from a virulent H5N1 challenge [19]. In Indonesia over twenty different vaccines intended for control of HPAIV H5N1 have been registered and the majority of commercial layers are vaccinated [4]. However, as is the case in other countries where HPAIV H5N1 is endemic, the field effectiveness of these vaccinations remains unknown [5, 21].

The objective of this study was to evaluate through longitudinal sampling, the effectiveness of H5N1 vaccination in small to medium-sized commercial layer flocks in Indonesia. These flocks belong to the “Sector 3” classification of poultry production [22] in which birds are kept under variable biosecurity and husbandry conditions and vaccinated with different AI vaccines. The results showed that HPAI vaccination had highly variable outcome and did not provide sufficiently long protective immunity in the majority of flocks.

## Material and methods

### Selection of commercial poultry for longitudinal surveillance

This was a prospective longitudinal study wherein individual birds from sixteen commercial layer flocks from Sector 3 in the provinces of West Java (WJ) and Special Region of Yogyakarta (DIY) were repeatedly sampled at regular intervals after 18 weeks of age (wo). Protocols for farms selection have been detailed elsewhere [22]. In brief, an initial visit was made between May and August 2012 to thirty-nine layer farms in the province of WJ and twenty-one layer farms in DIY and farm/flock data obtained. Sector 3 layers were targeted because of sizable population (from total layer population of 600 million approximately 100 million of layers are in Sector 3) (1), most farms practiced H5N1 vaccination and was possible to gain access to farms and flocks’ data for the conduct of the study. Commercial poultry Sector 1 and 2 also practice H5N1 vaccination, however access to both Sectors is restricted and flock data often treated as confidential. Farms in WJ were selected by the local District Veterinary Offices (through which access to farms was only possible) and in DIY by one of the authors (MW), who also facilitated enrolment of the poultry farms. From the data obtained during the initial visit, eight layer farms in WJ and eight in DIY were selected for the study based on the following criteria: (i) owners were willing to participate in a nine-month long study and allowed tagging of individual birds; and (ii), farms were representative of the Sector with respect to farm sizes, vaccines used, number of vaccination and husbandry practices. Sampling of selected flocks occurred between December 2012 and May 2014.

### Longitudinal surveillance of selected flocks

At the commencement of the study 25 birds were selected at random from within a flock housed in cages in one shed, tagged and placed into individual cages positioned uniformly across the entire shed area. At 18 wo and afterwards when the enrolled flock was 28, 38, 48, 58 and 68 wo, blood samples were collected, placed on ice and transported immediately to laboratories in Bogor or Yogyakarta. About one ml of blood was drawn by venipuncture of the brachial vein according to the Food and Agriculture Organisation protocol (http://www.fao.org/docrep/005/ac802e/ac802e0a.htm). Serum was removed and stored at 4°C if testing was to be accomplished within 48 hours, otherwise the sera were stored at -20°C.

### Haemagglutination and haemagglutination inhibition (HI) test

Haemagglutinating antigens (HA) were prepared and HI tests performed according to the protocols described by the World Organization for Animal Health (OIE) [23] using 4 HA units. To minimise variability of the HI testing performed in the two laboratories, one batch of HA antigen was used for testing of all sera. The HA antigens were back-titrated and reference positive sera of known titre and negative control sera were included in each test plate. Sera collected from the WJ flocks were tested for antibodies against A/chicken/West Java /Subang-29/2007 (Sb29), Pwt and A/chicken/Indonesia/BL/2003 (BL03) HA antigens and for the DIY flocks, the sera were tested against Sb29, Pwt and Skh HA antigens (S1 Table).

### Vaccines

Seven commercial AI vaccines were used by farms for vaccination of flocks that participated in the longitudinal study (S2 Table).

### Experimental infection of vaccinated layers with H5N1

A challenge study was conducted at the conclusion of the longitudinal study in which 22 birds from the Csa flock (Experiment 1) and 24 from the Spu flock (Experiment 2), at 70 wo, were moved from the farms into negative pressure isolators located within the biosafety level 3 experimental facility of the Indonesian Research Centre for Veterinary Science. Each bird was inoculated orally with 10^5^ median egg-infective doses of H5N1 strain Sb29. All birds were bled by venipuncture of the brachial vein (approximately one ml of blood/bird) for collection of sera just before and also at 7 and 14 days post-infection (dpi). Cloacal swabs were also collected at 3 and 7 dpi and used for virus isolation. Birds were observed four to five times during the day for clinical signs and discomfort such as ocular and nasal discharges, coughing, snicking and dyspnoea, swelling of the sinuses, listlessness, ruffled feathers, reduction in feed and water intake, cyanosis, nervous signs and diarrhea [23]. Based on clinical signs individual birds were scored as either normal, slightly ill/mildly depressed, ill/depressed and severely ill [24]. The intention was that following the diagnosis severely ill chickens be immediately euthanized by injection of sodium pentobarbital. Groups of unvaccinated control Csa and Spu birds were not available as unvaccinated birds are a significant risk factor for maintenance of H5N1 infections in vaccinated flocks and therefore could not be kept on commercial poultry farms. However the Sb29 H5N1 virus used for challenge was previously shown to induce 100% of mortality between 3 and 5 dpi in non-vaccinated commercial layers housed in the same biosafety level 3 experimental facility [25].

### Re-isolation of challenge virus

Isolation of Sb29 H5N1 virus from individual swab samples from the experimentally infected birds was performed according to the OIE Manual of Diagnostic Tests and Vaccines [23] and as previously described [25].

### Animal ethics approval

Challenge experiments were approved by the Animal Experimentation Ethics Committee of the Indonesian Research Institute for Veterinary Sciences, approval number BB/V/A/01/2013. The approved experimental protocols carried out were in accord with the standard procedures described in the OIE Manual of Diagnostic Tests and Vaccines [23] and stipulated that chickens could die following H5N1 challenge and that animals would be euthanized if severe clinical symptoms [24] were observed by a supervising veterinary pathologist specialising in poultry diseases.

### Statistical analyses

Three types of statistical analysis were performed: (i) Repeated measures analysis of variance was used to compare HI antibody titres in vaccinated layers form the longitudinal study. For the analysis birds that took part in the longitudinal study were grouped by farm and HI titres plotted for individual birds as a function of week of age. The timing of HPAI vaccination events for each farm was then superimposed on each plot as vertical lines. Three distinct groups of vaccination regimes/farms/flocks were evident: regime A, those vaccinated two to three times before 19 weeks of age; regime B, those vaccinated twice before 19 weeks of age with a single follow-up vaccination at around 40 weeks and regime C, farms that fitted neither of the above two categories. A repeated measures analysis of variance was performed and HI titres between and within regime A, B, and C groups compared using the "nlme" [26] and “multcomp” [27] packages in R (R Development Core Team, 2015). In brief, regime A (*n* = 8), B (*n* = 2) and C (*n* = 6) farms were selected in turn and analyses carried out to test the null hypothesis that individual bird Sb29 HI titres did not vary across farms within a regime group. Mauchly’s test of sphericity on each of the three data sets was significant indicating that the variances of the differences in Sb29 HI titres at different ages were not equal. To deal with this violation of one of the assumptions of a repeated measures analysis of variance, a multilevel model was developed for each regime group with Sb29 HI titre as the outcome, farm and age as explanatory variables and individual bird as a random effect. Within the multilevel model the assumption of sphericity was relaxed by specifying an unstructured covariate matrix. A multilevel model was then developed using test details from all birds from all sixteen farms, with an identical structure to the model described above. *A posteriori* contrasts were carried out to test the hypothesis that: (i) Sb29 HI titres from birds from regime A farms differed from birds from regimes B and C farms, and (ii) Sb29 HI titres from birds from regime B farms differed from birds from regime C farms. (ii) The Student’s *t*-test was used to compare the results obtained in the challenge study with confidence interval (CI) for the difference set at 95%. Comparisons were: (i) individual Sb29 HI titres at the time of challenge (day 0) in surviving versus birds that died; (ii) HI titres at 0 day in surviving Spu versus Spu birds that died; (iii) HI titres in surviving Csa birds at day 0 versus day 14, and (iv) mean death time (days) between Csa and Spu birds. (iii) Fisher’s exact test was used to compare proportions of infected birds and dead birds for the Csa and Spu groups. A value of p ≤ 0.05 was considered statistically significant.

## Results

### Relatedness of HA antigens used to measure AI immunity

Since layer flocks in the study were vaccinated with a variety of vaccines, four H5N1 strains, BL03, Pwt, Sb29 and Skh, isolated in Indonesia between 2004 and 2013 were used as HA antigens. Homologous HI titre for Sb29 was 5log2 and for BL03, Pwt and Skh antigens 7log_2_ (in the range detected for other H5N1 strains [7]), but their heterologous HI titres differed with all other HA antigens, indicating considerable antigenic differences between strains (S1 Table).

### Profile of study farms and flock vaccination

Eight commercial layer farms in the province of WJ and eight in the province of DIY took part in the longitudinal study. Farm data, vaccine used and vaccination schedules are shown in Table 1. Of the seven different vaccines, Medivac was the most frequently used (7/16 farms). Vaccines were based on seed strains from either the H5N1, H5N2 or H5N9 subtype. Although flocks were vaccinated between two and six times, the vaccination schedules differed on all farms (Table 1). All flocks were vaccinated before 19 wo (taken as the “point-of-lay”) either two, three or four times and 8/16 flocks were also re-vaccinated during the laying period. The most frequently used vaccination regime was three times before 19 wo. This regime was applied on six farms using five different vaccines.

**Table 1:** Profile of layer farms participating in the longitudinal surveillance study.

Province^a^	Farm/ flock^b^	District	Total birds (no of sheds)	Flock age at vaccination (weeks)	Vaccine used^c^ (age at vaccination in weeks)
**WJ**	Cwi	Cianjur	150,000 (50)	4, 12, 22, 44	Vaksimune AI
** **	Csa	Cianjur	97,000 (66)	1, 4, 13, 17, 35, 46	Medivac AI
	Cha	Cianjur	80,000 (18)	1, 5, 17	Caprivac AI-K
** **	Cci	Cianjur	100,000 (43)	1, 6, 19, 28, 45	Medivac [1, 6, 9] & Caprivac [28, 45]
	Ckr	Cianjur	55,000 (24)	1, 5, 9, 19, 29	Caprivac AI-K
** **	Spu	Sukabumi	12,000 (8)	5, 10, 17	Medivac AI
	Sta	Sukabumi	15,000 (25)	6, 16, 47	Medivac AI
** **	Ssc	Sukabumi	80,000 (24)	3, 12, 19, 28, 48	BioTek H5N2 [3,12,19] & BirdCLOSE [28, 48]
**DIY**	SL.1	Sleman	27,000 (13)	1, 11, 18	ProTek AI
	SL.2	Sleman	30,000 (10)	4, 8, 17	Vaksimun AI
** **	SL.3	Sleman	100,000 (38)	4, 9, 18	Gallimune™
	SL.4	Sleman	80,000 (30)	4, 14	Medivac AI
** **	SL.6	Sleman	30,000 (16)	6, 14, 40	Medivac AI
	SL.7	Sleman	20,000 (15)	1, 5, 18	Medivac AI
** **	KP.1	Kulon Progo	32,000 (21)	4, 17	Caprivac AI-K
	GK.1	Gunung Kidul	50,000 (30)	5, 13, 26, 40	ProTek AI

^a^ Provinces of West Java (WJ) and Special District of Yogyakarta (DIY).

^b^ Farm designation used in order to maintain farms’ anonymity, derived from abbreviations for the district in which the farm was located and the farm name.

^c^ Manufacturers’ designation. Conventional inactivated vaccines.

### Antibody titres in vaccinated layers determined using Sb29 HA antigen

**Fig 1:**
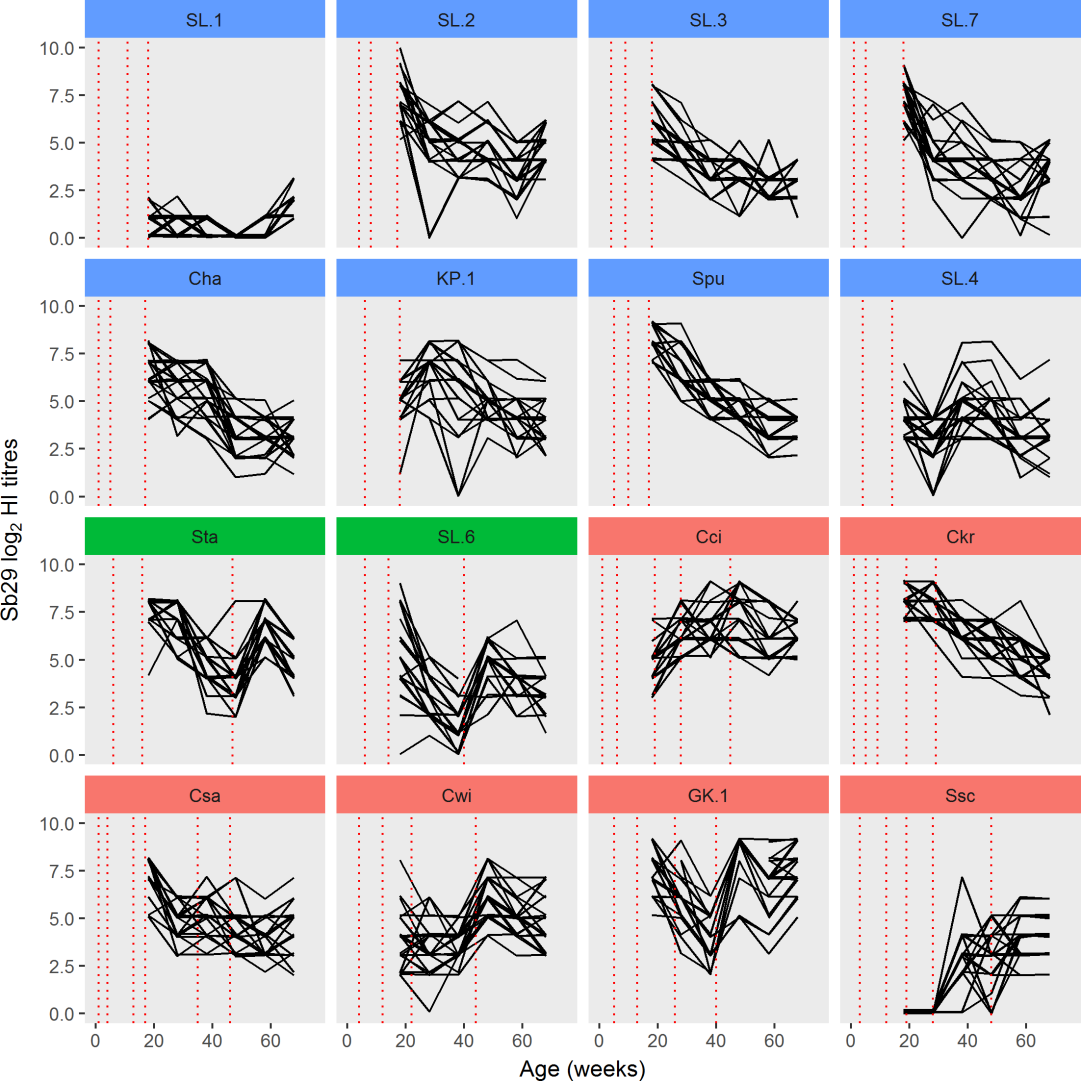
Haemagglutination inhibition (HI) titres against Sb29 H5N1 haemagglutinating antigen in vaccinated birds. Titres in individual birds determined at 18, 28, 38, 48, 58 and 68 weeks of age and grouped by flock/farm (*n* = 25 per farm) and vaccination regimes. Regime A–(blue) birds vaccinated two (SL.4 and KP.1) or three times (SL.1, SL.2, SL.3, SL.7, Cha and Spu) before 18 weeks of age; regime B (green)—birds vaccinated two times before 18 weeks of age, with a single follow-up vaccination at either 47 or 40 weeks (Sta and SL.6); regime C (red)–birds vaccinated two, three or four times before 18 weeks of age, with two or three follow-up vaccinations between 19 and 48 weeks of age. The timing of highly pathogenic avian influenza vaccination events for each farm are shown as dashed vertical lines.

A line plot showing Sb29 HI titres for individual birds, grouped by farm/flock/regime, as a function of age, is shown in Fig 1. Three distinct regime groups were evident. Flocks in regime A were vaccinated either two or three times before 19 wo. Those in the regime B group were vaccinated twice before 19 wo, with a single follow-up vaccination at 40 or 47 wo. Those in the regime C group were vaccinated two, three or four times before 19 wo with two or three follow-up vaccinations between 19 and 48 wo. In birds on Ssc and SL.1 farms, although they had been vaccinated two and three times, there were no detectable HI antibodies at 18 wo (Fig 1). The Ssc flock was re-vaccinated, whereas the SL.1 farm did not re-vaccinate and the flock remained HI antibody negative. With the exception of SL.4, there was a decline of HI antibody titres after 18 wo in all regime A flocks.

After adjusting for the effect of farm, week of sampling and individual bird-level effects, the Sb29 HI titres for regime A flocks were significantly lower (p < 0.01) compared with titres in regime B and C flocks (*z* test statistic -4.034; p < 0.01). Regime B flocks titres were lower than those in regime C flocks (*z* test statistic 1.949; p = 0.05). Exclusion of the SL.1 (non-responder) flock in this comparison did not alter these inferences. The Sb29 HI titres for flocks in the regime A (F_7,193_ = 107.191; p < 0.01), regime B (F_1,48_ = 164.49; p < 0.01) and regime C (F_5,144_ = 162.68; p < 0.01) groups were significantly different indicating that vaccination outcomes for flocks within each regime group were variable.

In the majority of flocks that responded to vaccination (10/15) all birds had similar Sb29 HI titres through the observation period (Fig 1), with coefficient of variation (that describes the level of variability within each flock) between 10 and 34. In five flocks (Cwi, Ssc, SL.4, SL.6 and SL.7) the Sb29 HI titre of individual birds differed at two to four time points with a coefficient of variation between 36 and 71 (S3 Table). In the SL.4 flock 16/25 birds had Sb29 HI titres that did not change significantly over the follow up period, but in nine birds Sb29 HI titres were higher at 38 wo (2–3 log_2_) in comparison to titres at 28 wo.

### Level and duration of protective antibody titres in vaccinated layers

**Fig 2:**
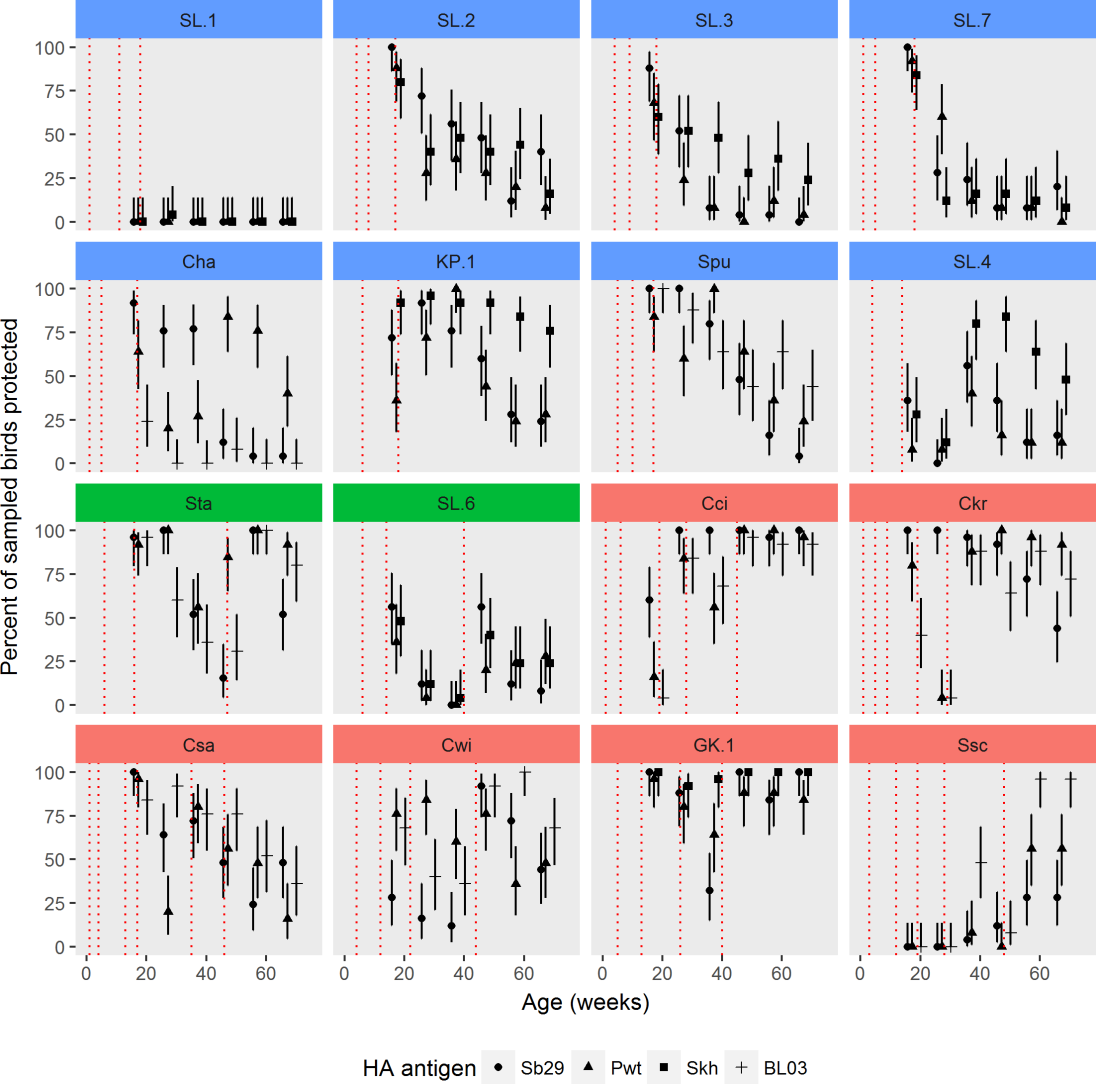
Proportion of vaccinated birds with protective haemagglutination inhibition (HI) titres of ≥4log2. HI titres in sera of individual vaccinated birds determined against Sb29, Pwt, Skh and BL03 H5N1 haemagglutinating antigen at 18, 28, 38, 48, 58 and 68 weeks of age and grouped by flock/farm (*n* = 25 per farm) and vaccination regimes A (blue), B (green) and C (pink) as detailed in Fig 1. The timing of highly pathogenic avian influenza vaccination for each farm are shown as dashed vertical lines. Percent of infected birds presented as proportion on the scale 0–1.0 (= 0–100%).

The percentage of birds with Sb29 HI titres of ≥4log_2_ that are considered protective [28], is shown in Fig 2. At 18 wo, in the majority of flocks (12/16) 60% - 100% of birds had Sb29 HI titres above 4log_2_ but in four flocks HI titres were below protective 4log_2_ level in spite of two or three vaccinations. The duration of protective immunity was variable and, to a degree, related to vaccination regime. From 18 wo and until 68 wo the number of protected birds declined in most flocks and only two flocks, Cci and GK.1, in regime C remained protected until 68 wo (Fig 2). Another two flocks (Ckr and Sta) in regime C and B, were protected until 58 and 48 wo, respectively. In comparison, in regime A, 4/8 flocks were protected at 38 wo, 2/8 only at 18 wo and 2/8 where not protected at any time. The overall short duration and a sudden drop of protective HI titres was not related to the vaccine used, nor the timing of vaccination, as could be concluded from a comparison of SL.3, SL.7 and Spu flocks. Re-vaccination during the laying period was efficacious when carried out in the presence of low antibody levels (Sta, SL.6 and Cwi flocks) and in some cases also when flocks were fully immune (Cci, Ckr and GK.1). Re-vaccination was not effective in other flocks (SL.6, Csa and Cwi), although birds had low antibody levels (Fig 2).

### Protective antibody titres in vaccinated layers determined by additional HA antigens

In addition to Sb29 HI titres, protective antibody titres were determined using Pwt and BL03 and Pwt and Skh HA antigens in WJ and DIY flocks, respectively (Fig 2). The percentage of protected birds obtained by three different HA antigens was similar in the majority of flocks (11/16), indicating that the Sb29 was an effective HA antigen for detecting vaccinal immunity, regardless of vaccine used. In five flocks (Ssc, KP.1, Cha, Cwi and SL.4) titres obtained by three HA antigens differed. In the Ssc flock the BL03 HI antibodies were the highest, as expected, since they increased following primary and secondary vaccination with the BirdCLOSE vaccine based on H5N1 strain homologous to BL03. In the KP.1 flock Skh HI titres remain high after 48 wo whereas Sb29 and Pwt titres declined, possibly due to vaccination or field challenge with Skh-related strains before 18 wo. In the Cha and Cwi flocks HI titres for Sb29, Pwt and Skh were inconsistent or contradictory. For example in the Cwi flock, Sb29 and BL03 titres increased following vaccination at 42 wo but not following vaccination at 22 wo, whereas Pwt titres remained high throughout the follow up period. In the SL.4 flock Sb29, Pwt and Skh HI titres were the same only in tree birds, whereas in others Pwt and Skh titres, in particular, were variable and inconsistent (results for six birds are shown in S1 Fig).

### Protective immunity in vaccinated layers following virulent H5N1 challenge

Birds from the Spu (regime A) and Csa (regime C) flocks vaccinated three and six times, were challenged at 70 wo with Sb29 H5N1 strain to determine whether birds from these groups differed in susceptibility to virulent H5N1 challenge (S4 Table), as predicted by their HI titres (Fig 2). Following challenge 75% and 50% of the Spu and Csa birds died between day 5 and 10 after challenge (mean death time of 7.5 days in both groups), but the difference in the mortality rate was not statistically significant. Clinical signs of depression and respiratory distress were visible in some birds, but only after 6 dpi and were scored as mild or moderate. The clinical signs observed, however, were not a reliable indicator if birds were to survive or die, and all deaths were sudden. The number of infected birds, 92% and 55% in Spu and Csa group, respectively, was significantly different (p < 0.05). The majority of Spu and Csa birds that survived infection, 5/6 and 10/11 respectively, had at the time of infection Sb29 Hi titres of ≥4log_2_ and the majority of those that died, 17/18 and 10/11 respectively, HI titres of ≤3 log_2_. Geometric mean Sb29 HI titre in surviving Spu and Csa birds was 3.8 and 4.3 log_2_ and statistically significantly different from the geometric mean Sb29 HI titres in birds that died, 2.7 and 2.7 log_2_, respectively (p < 0.001).

## Discussion

Seven different commercial AI vaccines were used for vaccination of sixteen flocks but none was consistently effective. Most of the vaccines were of H5N1 subtype, based either on A/chicken/Legok/2003, A/chicken/West Java/ 30/2007 or A/chicken/West Java/Pwt-Wij/2006 (Pwt), HPAIV strains that belong to clades, 2.1.1 and 2.1.3.2 and differed antigenically [10]. However it was not attempted to confirm the strain identity in any of the vaccine, for example by sequencing [8]. Since introduction of vaccination, over twenty vaccines have been registered for use in Indonesia [1, 8]. In 2011, the Ministry of Agriculture implemented regulations requiring that H5N1 HPAI vaccines use seed strain based on local H5N1 variants. This regulation permitted the sale of existing stock of imported vaccine, and this explains why several farms in our study used H5N2 and H5N9 based vaccines. The protective capacity of vaccines derived from H5N2, H5N9, A/chicken/Legok/2003 and A/chicken/West Java/Pwt-Wij/2006 (Pwt) strains has been established in laboratory experiments [8, 24] however many factors that influence vaccine effectiveness [18, 29] including application at farm level, are difficult to control in Indonesia [1].

The timing of flock vaccination differed on all farms, but three vaccination regimes/schedules were evident: regime A, in which flocks were vaccinated either two or three times before onset of lay at 19 wo, was the most commonly practiced; regime B, where flocks were vaccinated twice before 19 wo with a single follow-up vaccination at 40 or 47 wo and regime C where flocks were vaccinated two, three or four times before 19 wo with two or three follow-up vaccinations between 20 and 48 wo. There is no single recommended regime for HPAI vaccination of commercial poultry in the endemic situation [18, 20]. Two vaccinations prior to and one during lay, similar to regime B, have been proposed for control of low pathogenic avian influenza [30]. This regime has been also advocated for use in vaccination for HPAI [1, 31]. The manufacturer’s recommendation for Caprivac vaccine was similar to regime B whereas recommendations for Medivac vaccine were similar to either regime A or C. Although 9/16 flocks used Caprivac and Medivac vaccines, only in one flock was Medivac’s recommended vaccination protocol followed.

The field effectiveness of vaccination, as determined by the presence and titres of Sb29 HI antibodies, differed in all flocks and vaccination outcome was difficult to predict, except in general terms. In two flocks, no HI antibodies were detected in spite of the layer birds receiving two or three vaccinations prior to 19 wo, thus indicating undetected vaccine administration problems on these farms. Maternal antibodies did not appear to have played a major role in development of vaccinal immunity because all flocks were vaccinated at, or after 3 wo (and once or twice thereafter) at the time when AIV maternal antibodies are expected to be at low levels [25] and do not interfere with vaccination [32]. Antibody response was detected in the majority of vaccinated flocks and overall the level of Sb29 HI antibodies correlated with the number of vaccinations: In flocks vaccinated with regime C, HI titres were significantly higher through the observation period in comparison to HI titres in flocks vaccinated with regimes A and B. Similarly, HI titres in flocks vaccinated with regime B were higher than in those vaccinated with regime A. Thus the most frequently practiced H5N1 vaccination regime in Indonesia, regime A, induced the lowest level of vaccinal immunity. Importantly the HI titres of individual birds in each flock differed significantly from birds in other flocks vaccinated with the same regime, indicating that effectiveness of field vaccination was highly variable and farm related. A random cross-sectional survey of Sector 3 layer farms in the provinces of East and Central Java, undertaken following this study, confirmed that regime A (two to three vaccination before lay) was the most commonly practiced vaccination regime in Sector 3 layers (S.H. Irianingsih, personal communication).

An important characteristic of an effective H5N1 vaccination program is the number of birds that are protected from virulent challenge, i.e. the “level of flock immunity” [7, 33]. The current estimate of this is that where ≥60% of birds have HI titres of ≥ 4log_2_ spread of H5N1 challenge virus is reduced or prevented [33, 34]. The majority of vaccinated flocks in our study (12/16) had HI titres of ≥ 4log_2_ at 18 wo and thus were protected. However, thereafter, protective immunity declined at a variable rate and was associated with, to a degree, the number of vaccinations given. The regime A flocks were protected at between 18 and 38 wo, whereas those vaccinated with regime C were protected until 58 wo. Only two flocks, vaccinated with the regime C were fully protected until 68 wo. Sector 3 layers, including flocks in the study, are generally kept until between 80 and 100 wo [1, 22] and therefore the majority were not protected against H5N1 challenge throughout the production period.

There are only a few studies in which duration of HI antibodies following AI vaccination has been determined. Laboratory vaccination of commercial chickens by a single administration of an experimental H5N1 vaccine induced high titres of HI antibodies (approximately 8log_2_) that lasted up to 12 weeks [35], while in another study a single vaccination of free-range layers with H5N2 vaccine induced protective immunity that lasted for more than one year [36]. In studies with SPF chickens, a single dose of an inactivated H5N1 virus induced immunity that lasted up to 138 weeks [37] but in comparison infection with low-pathogenic live AI virus induced HI antibodies that lasted 22 weeks [38]. The reason for short duration and steep decline of HI antibodies in flocks surveyed is currently speculative, but could be due to insufficient antigenic mass in the inoculum [19]. Recommended vaccination dose for Indonesian vaccines vary between 0.3 ml and 0.5 ml per bird, injected either subcutaneously or intramuscularly, easy amenable to variation in volume administered considering that a large number of birds are vaccinated at any one time.

Re-vaccination during the laying period was important for maintaining the level of flock protective immunity, but it was not always effective even though ‘vaccine take’ and an anamnestic response in the order of ≥3log_2_ [39] was expected. In eight flocks that were revaccinated in total 14 times, increase in HI titres occurred only following six revaccinations, whereas in others, they declined or remained at the same level. Post-vaccination serological monitoring is not routinely undertaken in Sector 3 poultry in Indonesia and many poultry farms rely only on vaccine manufacturer recommendations to achieve flock-level H5N1 protection.

The Sb29 HA antigen, the nationally recommended antigen for measuring AI immunity during the study period, differed antigenically from BL03, Pwt and Skh. In spite of this, the Sb29 antigen was sufficiently cross-reactive and accurately measured immunity in the majority of vaccinated flocks, including those vaccinated with H5N2- and H5N9-based vaccines. In eleven flocks vaccinated with five different H5 vaccines there was an almost complete agreement between HI antibody titres, regardless of the HA antigen used. Studies have shown that H5N1 strains which are genetically similar may be antigenically different [10, 40] and complete antigenic homology between immunising or infecting strain and detecting HA antigen is rarely possible [40]. The primary and secondary immunisation with inactivated vaccines favour development of subtype specific antibodies and little cross-reaction is detected in HI test between the HA antigens and heterologous sera as was shown to be the case with Sb29, BL03, Pwt and Skh antigen using their primary antisera. Also this was evident in the Ssc flock where following two vaccinations with the BirdCLOSE vaccine HI titres obtained with homologous BL03 antigen were higher compared with Sb29 and Pwt HI titres. Multiple AIV immunisations are known to increase titres of both subtype-specific and cross-reactive HI antibodies, thus broadening the antigenic profile of induced antibodies and allowing for use of heterologous HA antigen for detection of AIV antibodies [41, 42].

That the Sb29 HI titres detected in vaccinated birds were an accurate measure of vaccinal immunity and correlated with protection was demonstrated in the challenge experiments of birds from two flocks vaccinated with regime A and C. It was predicted from the Sb29 HI titres at 68 wo that 79% and 48% of birds from of Spu and Csa birds, respectively, would be susceptible to virulent H5N1 challenge. Following challenge 92% and 75% of Spu and 54% and 50% of Csa birds were infected or died, respectively, confirming that the Sb29 measured HI titres reflected adequately the level of vaccinal and protective H5N1 immunity [7, 8, 34, 43].

Notably in all flocks, except for SL.4, there were no unexpected increases in HI titres in the absence of vaccination, thus suggesting the absence of field H5N1 challenge through the period of one year. This was an unexpected finding, contrary to anecdotal evidence that HPAIV H5N1 is common in Sector 3 poultry due to low biosecurity and ample opportunity for contacts with free range poultry, or wild birds. In the SL.4 flock in some birds (9/25) HI titres increased (2 -3log_2_) between 28 and 38 wo, an increase expected following revaccination (2 -3log_2_) rather then live virus challenge (5 - 8log_2_). Also highly inconsistent Pwt and Skh HI titres and high CV(%) indicated that SL.4 sera themself were the source of erroneous HI results [38].

## Conclusions

HPAI vaccination, intensively applied in Sector 3 layers in Indonesia, had highly variable outcome, including vaccination failures and did not provide sufficiently long protective immunity in the majority of flocks. Indonesia adopted HPAI vaccination in 2004 with the aim of reducing the incidence of H5N1 infections in poultry, with the ultimate objective of achieving eradication of the virus. Assessment of field effectiveness of the currently applied H5N1 vaccination was useful in demonstrating that vaccination, as practiced in Sector 3 poultry, could be improved. In particular, we have identified that the most frequently used vaccination regime, consisting of three vaccinations before 19 wo, does not provide sufficiently long lasting immunity and protection of layers with any of the commonly used HPAI vaccines. Instead, four or five vaccinations, of which two are during the laying period at 26–28 and 40–48 wo, would ensure longer lasting protection and further reduce the risk from exogenously introduced H5N1 infections. Monitoring the level of immunity in vaccinated flocks would help to identify key factors that contribute to inadequate responses to vaccination, short duration of protective immunity and vaccination failures. The timing of re-vaccination could be adjusted according to the flock immunity, ensuring an effective response and longer lasting protective immunity.

## Supporting information

S1 Table Antigenic relatedness between the haemagglutinating (HA) antigens used in the study determined by haemagglutination inhibition (HI) test. (DOC)

S2 Table Manufacturer’s information concerning AI vaccines and their recommended use. (DOC)

S3 Table Geometric mean Sb29 HI antibody titre and coefficient of variation in fifteen flocks that responded to vaccination. (DOC)

S4 Table Susceptibility of Spu birds vaccinated three times and Csa birds vaccinated six times with Medivac H5N1 vaccine to challenge with A/chicken/wj/Subang-29/2007 (Sb29) H5N1 strain at 70 weeks of age. (DOC)

S1 Fig Haemagglutination inhibition (HI) titres determined by Sb29, Pwt and Skh HA antigens in SL.4 birds vaccinated twice before 19 weeks of age with an HPAI vaccine. HI titres in bird no (i) to (vi) determined at 18, 28, 38, 48, 58 and 68 weeks of age.
(PPTX)

## References

[1] Sawitri Siregar E, Darminto, Weaver J, Bouma A. The vaccination programme in Indonesia. Dev Biol (Basel). 2007;130:151–158. 18411946

[2] Smith GJD, Naipospos TSP, Nguyen TD, De Jong MD, Vijaykrishna D, Usman TB, et al Evolution and adaptation of H5N1 influenza virus in avian and human hosts in Indonesia and Vietnam. Virology. 2006;350:258–268. doi: 10.1016/j.virol.2006.03.048 16713612 10.1016/j.virol.2006.03.048

[3] Yupiana Y, de Vlas SJ, Adnan NM, Richardus JH. Risk factors of poultry outbreaks and human cases of H5N1 avian influenza virus infection in West Java province, Indonesia. Int J Infect Dis. 2010;14(9):e800–e5. doi: 10.1016/j.ijid.2010.03.014 20637674 10.1016/j.ijid.2010.03.014

[4] Domenech J, Dauphin G, Rushton J, McGrane J, Lubroth J, Tripodi A, et al Experiences with vaccination in countries endemically infected with highly pathogenic avian influenza: the Food and Agriculture Organization perspective. Rev Sci Tech (International Office of Epizootics). 2009;28(1):293–305. 10.20506/rst.28.1.1865 19618633

[5] Swayne DE, Pavade G, Hamilton K, Vallat B, Miyagishima K. Assessment of national strategies for control of high-pathogenicity avian influenza and low-pathogenicity notifiable avian influenza in poultry, with emphasis on vaccines and vaccination. Rev Sci Tech (International Office of Epizootics). 2011;30(3):839–870. 10.20506/rst.30.3.2081 22435196

[6] Poetri ON, Bouma A, Murtini S, Claassen I, Koch G, Soejoedono RD, et al An inactivated H5N2 vaccine reduces transmission of highly pathogenic H5N1 avian influenza virus among native chickens. Vaccine. 2009;27(21):2864–2869. doi: 10.1016/j.vaccine.2009.02.085 19428896 10.1016/j.vaccine.2009.02.085

[7] Poetri ON, Van Boven M, Claassen I, Koch G, Wibawan IW, Stegeman A, et al Silent spread of highly pathogenic avian influenza H5N1 virus amongst vaccinated commercial layers. Res Vet Sci. 2014;97(3):637–641. doi: 10.1016/j.rvsc.2014.09.013 25301756 10.1016/j.rvsc.2014.09.013

[8] Swayne DE, Suarez DL, Spackman E, Jadhao S, Dauphin G, Kim-Torchetti M, et al Antibody titer has positive predictive value for vaccine protection against challenge with natural antigenic-drift variants of H5N1 high-pathogenicity avian influenza viruses from Indonesia. J Virol. 2015;89(7):3746–3762. doi: 10.1128/JVI.00025-15 25609805 10.1128/JVI.00025-15 PMC4403412

[9] Takano R, Nidom CA, Kiso M, Muramoto Y, Yamada S, Sakai-Tagawa Y, et al Phylogenetic characterization of H5N1 avian influenza viruses isolated in Indonesia from 2003–2007. Virology. 2009;390(1):13–21. doi: 10.1016/j.virol.2009.04.024 19464724 10.1016/j.virol.2009.04.024 PMC2861581

[10] Koel BF, van der Vliet S, Burke DF, Bestebroer TM, Bharoto EE, Yasa IWW, et al Antigenic variation of clade 2.1 H5N1 virus is determined by a few amino acid substitutions immediately adjacent to the receptor binding site. Mbio. 2014;5(3):e01070–e14. doi: 10.1128/mBio.01070-14 24917596 10.1128/mBio.01070-14 PMC4056550

[11] Hartaningsih N, Wibawa H, Pudjiatmoko, Rasa FST, Irianingsih SH, Dharmawan R, et al Surveillance at the molecular level: Developing an integrated network for detecting variation in avian influenza viruses in Indonesia. Prev Vet Med. 2015;120(1):96–105. doi: 10.1016/j.prevetmed.2015.02.015 25772529 10.1016/j.prevetmed.2015.02.015

[12] Wang J, Vijaykrishna D, Duan L, Bahl J, Zhang JX, Webster RG, et al Identification of the progenitors of Indonesian and Vietnamese avian influenza A (H5N1) viruses from southern China. J Virol. 2008;82(7):3405–3414. doi: 10.1128/JVI.02468-07 18216109 10.1128/JVI.02468-07 PMC2268469

[13] Lam TT-Y, Hon C-C, Lemey P, Pybus OG, Shi M, Tun HM, et al Phylodynamics of H5N1 avian influenza virus in Indonesia. Mol Ecol. 2012;21(12):3062–3077. doi: 10.1111/j.1365-294X.2012.05577.x 22574738 10.1111/j.1365-294X.2012.05577.x

[14] Bouma A, Muljono AT, Jatikusumah A, Nell AJ, Mudjiartiningsih S, Dharmayanti I, et al Field trial for assessment of avian influenza vaccination effectiveness in Indonesia. Rev Sci Tech (International Office Of Epizootics). 2008;27(3):633–642. 10.20506/rst.27.3.1823 19284033

[15] Bett B, McLaws M, Jost C, Schoonman L, Unger F, Poole J, et al The effectiveness of preventative mass vaccination regimes against the incidence of highly pathogenic avian influenza on Java island, Indonesia. Transbound Emerg Dis. 2015;62(2):163–173. doi: 10.1111/tbed.12101 23702277 10.1111/tbed.12101

[16] Ni Luh Putu Indi D, Hartawan R, Pudjiatmoko, Wibawa H, Hardiman, Balish A, et al Genetic characterization of clade 2.3.2.1 avian influenza A(H5N1) viruses, Indonesia, 2012. Emerg Infect Dis. 2014;20(4):671–674. doi: 10.3201/eid2004.130517 24656213 10.3201/eid2004.130517 PMC3966381

[17] Capua I. Vaccination for notifiable avian influenza in poultry. Rev Sci Tech (International Office of Epizootics). 2007;26(1):217–227. 17633304

[18] Spackman E, Swayne DE. Vaccination of gallinaceous poultry for H5N1 highly pathogenic avian influenza: current questions and new technology. Virus Res. 2013;178(1):121–132. doi: 10.1016/j.virusres.2013.03.004 23524326 10.1016/j.virusres.2013.03.004

[19] Swayne DE, Beck JR, Garcia M, Stone HD. Influence of virus strain and antigen mass on efficacy of H5 avian influenza inactivated vaccines. Avian Pathol. 1999;28(3):245–255. doi: 10.1080/03079459994731 26915380 10.1080/03079459994731

[20] van den Berg T, Lambrecht B, Marché S, Steensels M, Van Borm S, Bublot M. Influenza vaccines and vaccination strategies in birds. Comp Immunol, Microbiol Infect Dis. 2008;31(2–3):121–165. 17889937 10.1016/j.cimid.2007.07.004

[21] Sims LD. Lessons learned from Asian H5N1 outbreak control. Avian Dis. 2007;51(s1):174–181. 17494550 10.1637/7637-042806R.1

[22] Durr PA, Wibowo MH, Tarigan S, Artanto S, Rosyd MN, Ignjatovic J. Defining "Sector 3" poultry layer farms in relation to H5N1-HPAI- an example from Java, Indonesia. Avian Dis. 2016;60:183–190. doi: 10.1637/11134-050815-Reg 27309054 10.1637/11134-050815-Reg

[23] OIE. Avian Influenza. Manual of diagnostic tests and vaccines for terrestrial animals (mammals, birds and bees) Paris, France: Office International des Epizooties; 2008.

[24] Veits J, Römer-Oberdörfer A, Helferich D, Durban M, Suezer Y, Sutter G, et al Protective efficacy of several vaccines against highly pathogenic H5N1 avian influenza virus under experimental conditions. Vaccine. 2008;26(13):1688–1696. doi: 10.1016/j.vaccine.2008.01.016 18291561 10.1016/j.vaccine.2008.01.016

[25] Tarigan S, Indriani R, Durr PA, Ignjatovic J. Characterization of the M2e antibody response following highly pathogenic H5N1 avian influenza virus infection and reliability of M2e ELISA for identifying infected among vaccinated chickens. Avian Pathol. 2015;44(4):259–268. doi: 10.1080/03079457.2015.1042428 25915110 10.1080/03079457.2015.1042428

[26] Bates D, Pinheiro J. Computing REML or ML estimates for linear or nonlinear mixed-effects models. Comput Sci Stat. 1999;CONF 31:150.

[27] Hothorn T, Bretz F, Westfall P. Simultaneous inference in general parametric models. Biom J. 2008;50(3):346–363. doi: 10.1002/bimj.200810425 18481363 10.1002/bimj.200810425

[28] Ellis TM, Leung CYHC, Chow MKW, Bissett LA, Wong W, Guan Y, et al Vaccination of chickens against H5N1 avian influenza in the face of an outbreak interrupts virus transmission. Avian Pathol. 2004;33(4):405–412. doi: 10.1080/03079450410001724012 15370037 10.1080/03079450410001724012

[29] Mooney AJ, Tompkins SM. Experimental vaccines against potentially pandemic and highly pathogenic avian influenza viruses. Future Virology. 2013;8(1):25–41. doi: 10.2217/fvl.12.122 23440999 10.2217/fvl.12.122 PMC3579652

[30] Busani L, Toson M, Stegeman A, Pozza MD, Comin A, Mulatti P, et al Vaccination reduced the incidence of outbreaks of low pathogenicity avian influenza in northern Italy. Vaccine. 2009;27(27):3655–3661. doi: 10.1016/j.vaccine.2009.03.033 19464547 10.1016/j.vaccine.2009.03.033

[31] Peyre M, Fusheng G, Desvaux S, Roger F. Avian influenza vaccines: a practical review in relation to their application in the field with a focus on the Asian experience. Epidemiol Infect. 2009;137(1):1–21. doi: 10.1017/S0950268808001039 18700992 10.1017/S0950268808001039

[32] Abdelwhab EM, Grund C, Aly MM, Beer M, Harder TC, Hafez HM. Influence of maternal immunity on vaccine efficacy and susceptibility of one day old chicks against Egyptian highly pathogenic avian influenza H5N1. Vet Microbiol. 2012;155:13–20. doi: 10.1016/j.vetmic.2011.08.004 21920677 10.1016/j.vetmic.2011.08.004

[33] Bouma A, Claassen I, Natih K, Klinkenberg D, Donnelly CA, Koch G, et al Estimation of transmission parameters of H5N1 avian influenza virus in chickens. Plos Pathog. 2009;5(1):e1000281–e. doi: 10.1371/journal.ppat.1000281 19180190 10.1371/journal.ppat.1000281 PMC2627927

[34] Kumar M, Chu H-J, Rodenberg J, Krauss S, Webster RG. Association of serologic and protective responses of avian influenza vaccines in chickens. Avian Dis. 2007;51(1 Suppl):481–483. doi: 10.1637/7605-041706R1.1 17494614 10.1637/7605-041706R1.1

[35] Hwang SD, Kim HS, Cho SW, Seo SH. Single dose of oil-adjuvanted inactivated vaccine protects chickens from lethal infections of highly pathogenic H5N1 influenza virus. Vaccine. 2011;29:2178–2186. doi: 10.1016/j.vaccine.2010.12.013 21172378 10.1016/j.vaccine.2010.12.013

[36] Rudolf M, Pöppel M, Fröhlich A, Breithaupt A, Teifke J, Blohm U, et al Longitudinal 2 years field study of conventional vaccination against highly pathogenic avian influenza H5N1 in layer hens. Vaccine. 2010;28(42):6832–6840. doi: 10.1016/j.vaccine.2010.08.038 20727963 10.1016/j.vaccine.2010.08.038

[37] Sasaki T, Kokumai N, Ohgitani T, Sakamoto R, Takikawa N, Lin Z, et al Long lasting immunity in chickens induced by a single shot of influenza vaccine prepared from inactivated non-pathogenic H5N1 virus particles against challenge with a highly pathogenic avian influenza virus. Vaccine. 2009;27(38):5174–5177. doi: 10.1016/j.vaccine.2009.06.070 19596414 10.1016/j.vaccine.2009.06.070

[38] Meulemans G, Carlier MC, Gonze M, Petit P. Comparison of hemagglutination-Inhibition, agar gel precipitin, and enzyme-linked immunosorbent assay for measuring antibodies against Influenza viruses in chickens. Avian Dis. 1987;31(3):560–563. 2960313

[39] Swayne DE, Eggert D, Beck JR. Reduction of high pathogenicity avian influenza virus in eggs from chickens once or twice vaccinated with an oil-emulsified inactivated H5 avian influenza vaccine. Vaccine. 2012;30(33):4964–4970. doi: 10.1016/j.vaccine.2012.05.041 22652397 10.1016/j.vaccine.2012.05.041

[40] Fouchier RAM, Smith DJ. Use of antigenic cartography in vaccine seed strain selection. Avian Dis. 2010;54(s1):220–223. 20521635 10.1637/8740-032509-ResNote.1

[41] Abdelwhab EM, Grund C, Aly MM, Beer M, Harder TC, Hafez HM. Multiple dose vaccination with heterologous H5N2 vaccine: Immune response and protection against variant clade 2.2.1 highly pathogenic avian influenza H5N1 in broiler breeder chickens. Vaccine. 2011;29(37):6219–6225. doi: 10.1016/j.vaccine.2011.06.090 21745517 10.1016/j.vaccine.2011.06.090

[42] Khurana S, Chearwae W, Castellino F, Manischewitz J, King LR, Honorkiewicz A, et al Vaccines with MF59 adjuvant expand the antibody repertoire to target protective sites of pandemic avian H5N1 influenza virus. Sci Transl Med. 2010;2(15):15ra5–ra5. doi: 10.1126/scitranslmed.3000624 20371470 10.1126/scitranslmed.3000624

[43] Swayne DE, Beck JR, Perdue ML, Beard CW. Efficacy of vaccines in chickens against highly pathogenic Hong Kong H5N1 avian influenza. Avian Dis. 2001;45(2):355–365. 11417815

