# Supplementary material for: Field effectiveness of highly pathogenic avian influenza H5N1 vaccination in commercial layers in Indonesia
Source: PLoS One. 2018 Jan 10;13(1):e0190947. doi: 10.1371/journal.pone.0190947 (PMC5761929; doi:10.1371/journal.pone.0190947)
Supplement: S1 Table — (DOC) [file pone.0190947.s001.doc]

**S1 Table**. **Antigenic relatedness between the haemagglutinating (HA) antigens used in the study determined by haemagglutination inhibition (HI) test.**

|  | HI titres with antisera to ^a^ | | | |
| --- | --- | --- | --- | --- |
| HA antigen | BL03 | Pwt | Sb29 | Skh |
| A/chicken/Indonesia/BL/2003 (BL03) ^b^ | **128 (7)** ^d^ | 32 (5) | 16 (4) | 16 (4) |
| A/chicken/West Java/Pwt-Wij/2006 (Pwt) ^b^ | 32 (5) | **128 (7)** | 16 (4) | 32 (5) |
| A/chicken/West Java/Subang-29/2007 (Sb29) ^b^ | 2 (1) | 8 (3) | **32 (5)** | 2 (1) |
| A/duck/Sukoharjo/2012 (Skh) ^c^ | 16 (4) | 32 (5) | 2 (1) | **128 (7)** |

^a^ Reciprocal of the last dilution of sera (starting dilution ½) that inhibited haemagglutination of red blood cells by the HA antigen (log_2_ titre); HI titres of 2 (1log_2_) are considered negative and titres of above 16 (>4log_2_) as protective.

^b^ Indonesian H5N1 strains, clades 2.1.1 (BL03) and 2.1.3.2 (Pwt and Sb29). Strains BL03 (accession no AY651374) and Pwt (accession no EU124148.1) were approved for use as vaccine strains and Sb29 (accession no not available) for measuring vaccine induced immunity and protection.

^c^ Indonesian H5N1 strain clade 2.3.2.1 accession no KC417271. Approved for use as a vaccine strain.

^d^ Homologous titres are in bold.
