# Supplementary material for: Field effectiveness of highly pathogenic avian influenza H5N1 vaccination in commercial layers in Indonesia
Source: PLoS One. 2018 Jan 10;13(1):e0190947. doi: 10.1371/journal.pone.0190947 (PMC5761929; doi:10.1371/journal.pone.0190947)
Supplement: S2 Table — (DOC) [file pone.0190947.s002.doc]

**S2 Table. Manufacturer’s information concerning AI vaccines and their recommended use.**

| **Vaccine** | **Manufacturer** | **Seed strain and subtype** | **Recommendations for vaccination** |
| --- | --- | --- | --- |
| BioTek | PT Biotek, Indonesia | A/Turkey/England/N-28/73 (H5N2) | Not available |
| Bird CLOSE 5.1 | Shigeta Animal Pharmaceuticals Inc | Based on recombinant virus: HA gene derived from A/chicken/Legok/2003 (H5N1) (Genbank accession no GU05242600) | First vaccination at 10-12 day old with 0.5 ml per chick and revaccination within 3-5 weeks and follow up vaccination in the areas with risk of AI infection |
| Caprivac AI-K | Caprifarmindo, Bandung | A/chicken/West Java/Pwt-Wij/2006 (Pwt) (H5N1) (accession no EU124148.1) | By subcutaneous injection in the lower neck at 10 day old and intramuscular and subcutaneous injection at 8 and 40-42 week old |
| Gallimune**™** | Merial, Lyone, France | A/turkey/Wisconsin/1/1968  (H5N9) | Dose of 0.3 ml given to pullets |
| Medivac AI | Medion Farma Jaya, Bandung | A/chicken/West Java/Pwt-Wij/2006 (Pwt) (H5N1) | Administration at 10 day old and revaccination at 15 week old or at 9 and 17 week old in areas without, or with risk of AI infection, respectively, with subsequent vaccination at every 3-4 months based on titre evaluation |
| ProTek AI | PT Biotek, Indonesia | A/Turkey/England/N-28/73 (H5N2) | Administered 0.3 ml per chick 2-4 week old subcutaneously into the neck or 0.5 ml per chick ≥5 week old intramuscularly into breast muscle |
| Vaksimune AI | Pt Vaksindo Satwa Nusantra | A/chicken/West Java/ 30/2007 (H5N1), clade 2.1.3.2 (accession no KJ842543) (22) | Administration by subcutaneous or intramuscular injection |
