# Supplementary material for: Field effectiveness of highly pathogenic avian influenza H5N1 vaccination in commercial layers in Indonesia
Source: PLoS One. 2018 Jan 10;13(1):e0190947. doi: 10.1371/journal.pone.0190947 (PMC5761929; doi:10.1371/journal.pone.0190947)
Supplement: S3 Table — (DOC) [file pone.0190947.s003.doc]

**S3 Table. Geometric mean Sb29 HI antibody titre and coefficient of variation in fifteen flocks that responded to vaccination.**

|  |  | **Week of age** | | | | | | | | | | | |
| --- | --- | --- | --- | --- | --- | --- | --- | --- | --- | --- | --- | --- | --- |
| **HA Antigen** |  | **18** | | **28** | | **38** | | **48** | | **58** | | **68** | |
|  | **Flock** | **GMT^a^** | **CV^b^** | **GMT** | **CV** | **GMT** | **CV** | **GMT** | **CV** | **GMT** | **CV** | **GMT** | **CV** |
|  |  | **Log2** | **(%)** | **Log2** | **(%)** | **Log2** | **(%)** | **Log2** | **(%)** | **Log2** | **(%)** | **Log2** | **(%)** |
| **Sb29** | Cha^c^ | 6.4 | 20 | 5.6 | 22 | 5.4 | 23 | 3.2 | 34 | 3.4 | 29 | 2.8 | 33 |
|  | Spu | 8.3 | 10 | 5.1 | 14 | 5.1 | 14 | 4.6 | 26 | 3.4 | 27 | 3.4 | 21 |
|  | Sta | 7.5 | 12 | 7.1 | 16 | 4.6 | 22 | 3.8 | 31 | 6.7 | 13 | 4.7 | 21 |
|  | Cci | 4.7 | 22 | 6.3 | 15 | 6.6 | 16 | 7.1 | 20 | 6 | 22 | 6.2 | 13 |
|  | Ckr | 8 | 9 | 7.8 | 11 | 6.3 | 13 | 5.9 | 16 | 5.2 | 21 | 5.2 | 18 |
|  | Csa | 7.3 | 16 | 4.9 | 20 | 5.1 | 20 | 4.4 | 25 | 3.8 | 25 | 4.4 | 29 |
|  | Cwi | 3.8 | 40 | 3.3 | 41 | 3.5 | 23 | 6 | 19 | 5 | 19 | 4.6 | 30 |
|  | Ssc | 0 | N/A^d^ | 0 | N/A | 2.9 | 47 | 2.9 | 51 | 4 | 29 | 4 | 29 |
|  | SL.2 | 7.8 | 19 | 4.7 | 36 | 4.5 | 26 | 4.7 | 25 | 3.3 | 33 | 4.5 | 18 |
|  | SL.3 | 5.9 | 20 | 4.7 | 19 | 3.4 | 24 | 3.4 | 27 | 2.6 | 28 | 2.9 | 27 |
|  | SL.7 | 7.6 | 13 | 4.3 | 27 | 3.9 | 36 | 3.2 | 30 | 2.5 | 54 | 3.5 | 39 |
|  | KP.1 | 5 | 24 | 6.9 | 21 | 5.6 | 24 | 4.9 | 20 | 4.1 | 29 | 3.9 | 30 |
|  | SL.4 | 4.2 | 71 | 2.3 | 47 | 4.8 | 28 | 4.4 | 28 | 3.2 | 37 | 3.4 | 43 |
|  | SL.6 | 5.1 | 43 | 3 | 38 | 1.5 | 71 | 4.8 | 24 | 3.8 | 29 | 3.2 | 32 |
|  | GK.1 | 7.7 | 18 | 5.9 | 22 | 3.8 | 31 | 8.6 | 21 | 6.6 | 27 | 7.5 | 16 |

^a^GMT = Geometric mean titre (log2) of 25 birds.

^c^CV% = Coefficient of variation indicates the level of variability of HI titres within each flock. As individual bird HI titres ranged from 0 to 10, CV(%) is used as a standardised measure, obtained from the formulae CV(%) = 100 x StD/GMT, instead of standard deviation (StD) alone.

^c^The majority of flocks had CV of between 10 and 34, whereas five flocks, highlighted, had CV(%) of between 36 and 71 on at least two time points.

^d^N/A=Not applicable.
