# Supplementary material for: Field effectiveness of highly pathogenic avian influenza H5N1 vaccination in commercial layers in Indonesia
Source: PLoS One. 2018 Jan 10;13(1):e0190947. doi: 10.1371/journal.pone.0190947 (PMC5761929; doi:10.1371/journal.pone.0190947)
Supplement: S4 Table — (DOC) [file pone.0190947.s004.doc]

**S4 Table. Susceptibility of Spu birds vaccinated three times and Csa birds vaccinated six times with Medivac**

**H5N1 vaccine to challenge with A/chicken/wj/Subang-29/2007 (Sb29) H5N1 strain at 70 weeks of age**

| **Flock^a^** | **Bird No** | **VI at dpi^b^** | **Sb29 HI titrs (log2) in surviving birds at dpi^c^** | | | **Bird No** | **VI at dpi** | **Sb29 HI titres (log2) in birds that died at dpi^c^** | | **Time of death (dpi)** |
| --- | --- | --- | --- | --- | --- | --- | --- | --- | --- | --- |
|  |  |  | 0 | 7 | 14 |  |  | 0 | 7 |  |
| Spu | Spu-2 | 3 | 3 | 4 | 8 | Spu-1 | 7 | 3 | 4 | 10 |
|  | Spu-7 | 7 | 4 | 5 | 8 | Spu-3 | 3 | 3 | n/a | 5 |
|  | Spu-16 | - | 4 | 5 | 8 | Spu-4 | 3 | 3 | n/a | 6 |
|  | Spu-18 | - | 4 | 7 | 8 | Spu-5 | 3 | 3 | n/a | 6 |
|  | Spu-24 | 3, 7 | 4 | 4 | 8 | Spu-6 | 7 | 4 | 4 | 10 |
|  | Spu-25 | 7 | 4 | 3 | 8 | Spu-8 | 3, 7 | 3 | 5 | 8 |
|  |  |  | **3.8**^d^**,*** | **4.7**^d^ | **8**^d^ | Spu-9 | 3 | 0 | n/a | 6 |
|  |  |  |  |  |  | Spu-10 | 3 | 3 | 3 | 8 |
|  |  |  |  |  |  | Spu-11 | 3 | 2 | n/a | 6 |
|  |  |  |  |  |  | Spu-12 | 7 | 3 | 6 | 8 |
|  |  |  |  |  |  | Spu-13 | 3, 7 | 3 | 6 | 9 |
|  |  |  |  |  |  | Spu-15 | 3 | 3 | n/a | 6 |
|  |  |  |  |  |  | Spu-17 | 7 | 3 | n/a | 7 |
|  |  |  |  |  |  | Spu-19 | 7 | 3 | 4 | 10 |
|  |  |  |  |  |  | Spu-20 | 7 | 3 | 4 | 9 |
|  |  |  |  |  |  | Spu-21 | 7 | 3 | 3 | 9 |
|  |  |  |  |  |  | Spu-22 | 3 | 2 | n/a | 5 |
|  |  |  |  |  |  | Spu-23 | 3 | 2 | n/a | 7 |
|  |  |  |  |  |  |  |  | **2.7d, *** |  | 7.5^e^ |
| Csa | Csa-2 | - | 5 | 6 | 7 | Csa-4 | 3 | 3 | 4 | 10 |
|  | Csa-3 | 3 | 4 | 8 | 9 | Csa-5 | 3 | 3 | n/a | 5 |
|  | Csa-6 | - | 5 | 8 | 8 | Csa-10 | 3 | 3 | n/a | 6 |
|  | Csa-7 | - | 4 | 8 | 8 | Csa-12 | 3 | 3 | n/a | 6 |
|  | Csa-9 | - | 5 | 8 | 8 | Csa-13 | 3 | 4 | 5 | 10 |
|  | Csa-11 | - | 4 | 4 | 7 | Csa-15 | 7 | 3 | 6 | 8 |
|  | Csa-14 | - | 5 | 7 | 7 | Csa-16 | 3 | 0 | n/a | 6 |
|  | Csa-17 | - | 4 | 8 | 8 | Csa-18 | 3 | 3 | 3 | 8 |
|  | Csa-20 | - | 3 | 9 | 9 | Csa-19 | 3 | 2 | n/a | 6 |
|  | Csa-21 | - | 4 | 8 | 8 | Csa-22 | 3 | 3 | 6 | 8 |
|  | Csa-24 | - | 4 | 8 | 10 | Csa-23 | 3 | 3 | 6 | 9 |
|  |  |  | **4.2**^d^**, **** | **7.5**^d^ | **8.1**^d^ |  |  | **2.7**^d^,****** |  | 7.5^e^ |

^a^Vaccinated on farms and predicted from their Sb29 HI titres at 68 weeks of age that 79% and 48% of Spu and Csa birds, respectively, would be susceptible to virulent H5N1 challenge. At 70 weeks of age 24 Spu and 22 Csa birds transferred from farms to biosafety level 3 facility and inoculated orally with 10^5^ EID_50_ of A/chicken/wj/Subang-29/2007 (Sb29) H5N1.

^b^ VI, dpi= Virus isolation from cloacal swab at 3 and/or 7 days post-infection.

^c^ From Spu and Csa challenged birds 6/24 (75%) and 11/22 (50%) died, respectively, by 14 dpi.

^d^Log2 geometric mean HI titre.

e Mean death time of the group. Day 5 and 10

*p-Value < 0.05 in pairwise comparison of HI titres of Spu birds that survived and those that died.

**p-Value < 0.001 in pairwise comparison of HI titres of Csa birds that survived and those that died.
