# Supplementary figures and images for: Field effectiveness of highly pathogenic avian influenza H5N1 vaccination in commercial layers in Indonesia
Source: PLoS One. 2018 Jan 10;13(1):e0190947. doi: 10.1371/journal.pone.0190947 (PMC5761929; doi:10.1371/journal.pone.0190947)

## Slide 1
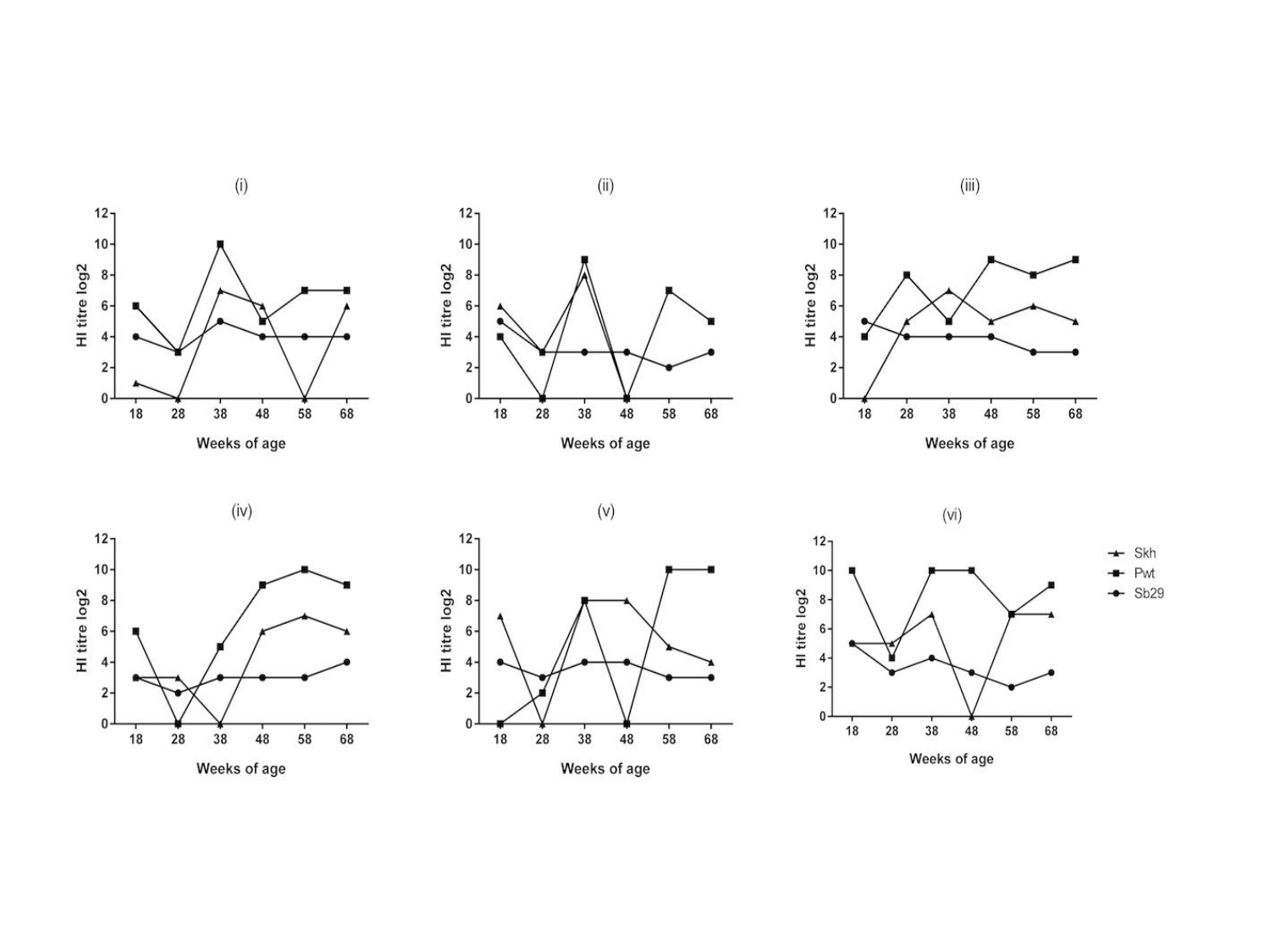

Supplement: S1 Fig — HI titres in bird no (i) to (vi) determined at 18, 28, 38, 48, 58 and 68 weeks of age. (PPTX) [file pone.0190947.s005.pptx]
